# Action of Carvacrol on *Parascaris* sp. and Antagonistic Effect on Nicotinic Acetylcholine Receptors

**DOI:** 10.3390/ph14060505

**Published:** 2021-05-26

**Authors:** Sasa M. Trailovic, Milan Rajkovic, Djordje S. Marjanovic, Cédric Neveu, Claude L. Charvet

**Affiliations:** 1 Department of Pharmacology and Toxicology, Faculty of Veterinary Medicine, University of Belgrade, 11000 Belgrade, Serbia; sasa@vet.bg.ac.rs (S.M.T.); milan.rajkovic.vet@gmail.com (M.R.); drdjolevet@yahoo.com (D.S.M.); 2 Department of Biology, Faculty of Veterinary Medicine, University of Belgrade, 11000 Belgrade, Serbia; 3 INRAE, Université de Tours, ISP, 37380 Nouzilly, France; cedric.neveu@inrae.fr

**Keywords:** *Parascaris*, carvacrol, nicotinic acetylcholine receptors, muscle contraction, electrophysiology, *Xenopus* oocytes, mode of action

## Abstract

*Parascaris* sp. is the only ascarid parasitic nematode in equids and one of the most threatening infectious organisms in horses. Only a limited number of compounds are available for treatment of horse helminthiasis, and *Parascaris* sp. worms have developed resistance to the three major anthelmintic families. In order to overcome the appearance of resistance, there is an urgent need for new therapeutic strategies. The active ingredients of herbal essential oils are potentially effective antiparasitic drugs. Carvacrol is one of the principal chemicals of essential oil from *Origanum*, *Thymus*, *Coridothymus*, *Thymbra*, *Satureja* and *Lippia* herbs. However, the antiparasitic mode of action of carvacrol is poorly understood. Here, the objective of the work was to characterize the activity of carvacrol on *Parascaris* sp. nicotinic acetylcholine receptor (nAChR) function both in vivo with the use of worm neuromuscular flap preparations and in vitro with two-electrode voltage-clamp electrophysiology on nAChRs expressed in *Xenopus* oocytes. We developed a neuromuscular contraction assay for *Parascaris* body flaps and obtained acetylcholine concentration-dependent contraction responses. Strikingly, we observed that 300 µM carvacrol fully and irreversibly abolished *Parascaris* sp. muscle contractions elicited by acetylcholine. Similarly, carvacrol antagonized acetylcholine-induced currents from both the nicotine-sensitive AChR and the morantel-sensitive AChR subtypes. Thus, we show for the first time that body muscle flap preparation is a tractable approach to investigating the pharmacology of *Parascaris* sp. neuromuscular system. Our results suggest an intriguing mode of action for carvacrol, being a potent antagonist of muscle nAChRs of *Parascaris* sp. worms, which may account for its antiparasitic potency.

## 1. Introduction

Helminth infections of livestock are of considerable importance and cause major financial losses [1]. *Parascaris* sp. is the largest nematode parasite of equids, representing a major threat in equine medicine. *Parascaris* sp. worms have a very high prevalence especially in foals with important impact in terms of morbidity and mortality [2,3]. The worms remain in the intestine of the equids and are targets for anthelmintic drugs. Only a limited number of compounds are available for treatment of horse helminthiasis with the macrocyclic lactones being the most recently developed drug class of veterinary anthelmintics, marketed since the 1980s [1,4]. Anthelmintic resistance is a major problem in veterinary medicine, and *Parascaris* sp. worms have recently developed resistance to the three major anthelmintic families [5,6,7,8,9]. In order to overcome the appearance of resistance, there is an urgent need for new therapeutic strategies, especially new chemical entities [1,10]. Based on their pharmacological properties, the active ingredients of herbal essential oils are potentially effective antiparasitic drugs [11,12,13]. Carvacrol and thymol are monoterpenic phenol isomers and the principal chemicals of essential oil from *Origanum*, *Thymus*, *Coridothymus*, *Thymbra*, *Satureja* and *Lippia* herbs [14]. Carvacrol is known for its wide use in traditional pharmacopeia due to antimicrobial and disinfectant properties [15,16,17]. In addition, some studies previously indicated that carvacrol has antinematodal properties against pathogenic helminths such as the pig roundworm *Ascaris suum* [18], the sheep parasite *Haemonchus contortus* [19,20], the fish parasite *Anisakis simplex* [14,21] and plant parasitic nematodes [22], and it can also kill the free-living model nematode *Caenorhabditis elegans* [23,24,25]. However, the antiparasitic mode of action of monoterpenoid compounds is poorly understood, and their potential use against horse parasites has not yet been investigated. Previous studies evidenced that the anthelmintic effect of carvacrol might be mediated through different ligand-gated ion channel subtypes including tyramine, acetylcholine and GABA receptors of nematodes [18,25,26,27,28] as well as acetylcholinesterase [14].

Acetylcholine is a major excitatory neurotransmitter in both vertebrates and invertebrates. The nicotinic acetylcholine receptors (nAChRs) are major targets for antinematodal drugs such as pyrantel and levamisole [29,30]. They are members of the cys-loop ligand-gated ion channel superfamily and consist of five subunits arranged around a central pore [31]. Despite the large diversity of nAChR subunit genes present in nematodes, few receptor subtypes have been characterized to date. Two nAChR subtypes have been described to mediate fast neurotransmission at the neuromuscular junction in the free-living model nematode *Caenorhabditis elegans* [32]: the levamisole-sensitive nAChR (L-AChR), which is a heteromeric ion channel made of five different subunits, and the prototypical nicotine-sensitive nAChR (N-AChR), which is composed of five identical subunits encoded by the *Cel-acr-16* gene [33,34]. In parasites, the ACR-16 receptor subunit was recently isolated and characterized from *Parascaris* sp. as well as the pig parasite, *Ascaris suum* [35,36]. When expressed in *Xenopus laevis* oocytes, ACR-16 formed a functional homomeric N-AChR, which is activated by nicotine. Furthermore, a new subtype of nematode AChR preferentially activated by morantel was reported in *Parascaris* sp. (M-AChR) along with the small ruminant parasite *Haemonchus contortus* [29]. Interestingly, parasitic nematodes affecting humans or animals possess two closely related AChR subunit genes that are essentially absent in free-living or plant parasitic species: *acr-26* encodes an alpha subunit, and *acr-27* encodes a non-alpha subunit. Hence, ACR-26 and ACR-27 subunits from *Parascaris* sp. were found to form a functional AChR when co-expressed in *Xenopus* oocytes, with higher affinities for pyrantel and morantel than for acetylcholine. Importantly, the heterologous expression of *Parascaris-acr-26* and *acr-*27 as transgenes in the model nematode *Caenorhabditis elegans* also drastically increased morantel and pyrantel sensitivity in vivo [29].

Here, the objective of the work was to characterize and investigate the activity of carvacrol at different concentrations on *Parascaris* sp. nicotinic acetylcholine receptors both for nAChR function in vivo with the use of worm neuromuscular flap preparations and in vitro for nAChRs expressed in *Xenopus* oocytes. Strikingly, we observed that carvacrol abolished *Parascaris* sp. muscle contraction elicited by acetylcholine. Likewise, carvacrol inhibited acetylcholine-induced currents on both N-AChR and M-AChR subtypes. Thus, we show carvacrol is a potent antagonist of muscle AChRs, which may account for its antiparasitic potency against *Parascaris* sp. worms.

## 2. Results

### 2.1. Acetylcholine-Induced Contraction of Parascaris sp. Body Muscle Flap Preparation

**Figure 1:**
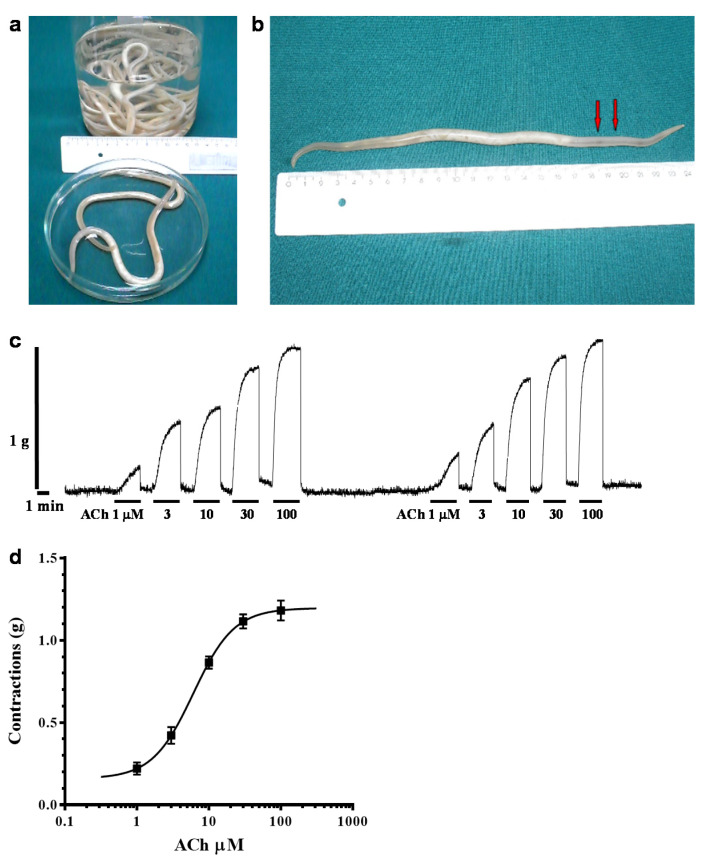
Contraction of *Parascaris* sp. muscle strips produced by acetylcholine. (**a**) Adult female *Parascaris* sp. collected from horses and used in this study; (**b**) photograph of a single worm indicating the location of the body muscle flap (1 cm length between the two red arrows), within the anterior part of the worm (3–4 cm caudal to the head), to be dissected for isometric contraction measurements; (**c**) isometric contractions of *Parascaris* sp. muscle flap produced by increasing concentrations of acetylcholine (ACh) from 1 to 100 µM (short bars); (**d**) concentration–response plot for ACh fitted with non-linear regression, with mean contraction in g ± SE (*n* = 5).

*Parascaris* sp. and *A. suum* worms are closely related ascarid species with similar anatomy and morphology (Figure 1a). The presence of acetylcholine receptors on *Parascaris* sp. muscles is anticipated as every nematode is supposed to synthesize acetylcholine and receptors, although this has not yet been functionally evidenced. Therefore, as for *A. suum*, it is expected that the application of acetylcholine on *Parascaris* sp. muscle strips would produce muscle contractions. As a first step, we adapted the muscle isometric contraction approach, which previously was used in *A. suum* studies [26,37]. Due to worm size differences, we had to modify the method of dissection. The part of the *Parascaris* worm that we dissected for contractions was 4 to 5 cm behind the head instead of 2–3 cm for *A. suum* (Figure 1b). In addition, in order to cause contractions after acetylcholine application, we had to use a larger initial tension (1.5 g). On the other hand, the maximal contractions were no higher than the contractions previously obtained in *A. suum* experiments [26,37]. As a result, we were able to measure contractions of nerve–muscle strip preparations induced by ACh. Figure 1c shows a representative recording of the *Parascaris* sp. muscle flap contractions produced by increasing concentrations of acetylcholine, while in Figure 1d we present a concentration–response plot for ACh fitted with non-linear regression. Increasing concentrations of ACh caused dose-dependent contractions of *Parascaris* sp. The control median effective concentration (EC_50_) of ACh was 6.08 µM (log EC_50_ = 0.78 ± 0.079, *n* = 5), while the maximal effect (R_max_) was 1.19 ± 0.051 g obtained with 100 µM ACh. Overall, these results indicate that *Parascaris* sp. body muscle flap preparation is an amenable approach for investigating the pharmacology of its neuromuscular system.

### 2.2. Carvacrol Abolishes Acetylcholine-Induced Contractions of Parascaris sp. Muscle Strips

**Figure 2:**
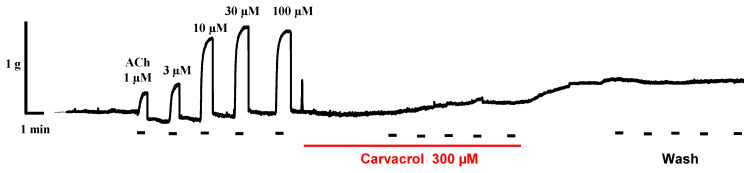
Effect of carvacrol on contractions of *Parascaris* sp. muscle strips produced by acetylcholine. Isometric contractions of *Parascaris* sp. muscle flap produced by increasing concentrations of acetylcholine (ACh) from 1 to 100 µM (left panel, short bars) and inhibition of contractions mediated by 300 µM carvacrol (middle panel, full line). Absence of ACh-induced response recovery after washing the preparation (right panel).

Previous studies highlighted the inhibitory effect of carvacrol on *A. suum* isolated muscle flap contractions caused by ACh [26,28]. In order to obtain first insights into the mode of action of carvacrol on *Parascaris* sp. worms, we determined the effect of carvacrol in isometric contractions of isolated segments of *Parascaris* sp. Figure 2 shows an inhibitory effect of carvacrol (300 µM) on the contractions of nerve–muscle preparation of *Parascaris* sp. induced by ACh. Strikingly, carvacrol completely abolished the contraction induced by ACh even at 100 µM, which is the highest concentration assessed and was used to achieve the maximal contraction effect. Interestingly, the inhibitory effect of 300 µM carvacrol remained even after removal of carvacrol from experimental baths. Altogether, our results show isometric contractions of *Parascaris* sp. muscle strips produced by increasing concentrations of ACh and full inhibition of contractions following application of carvacrol.

### 2.3. Effect of Carvacrol on the Parascaris sp. Morantel-AChR Expressed in Xenopus oocytes

**Figure 3:**
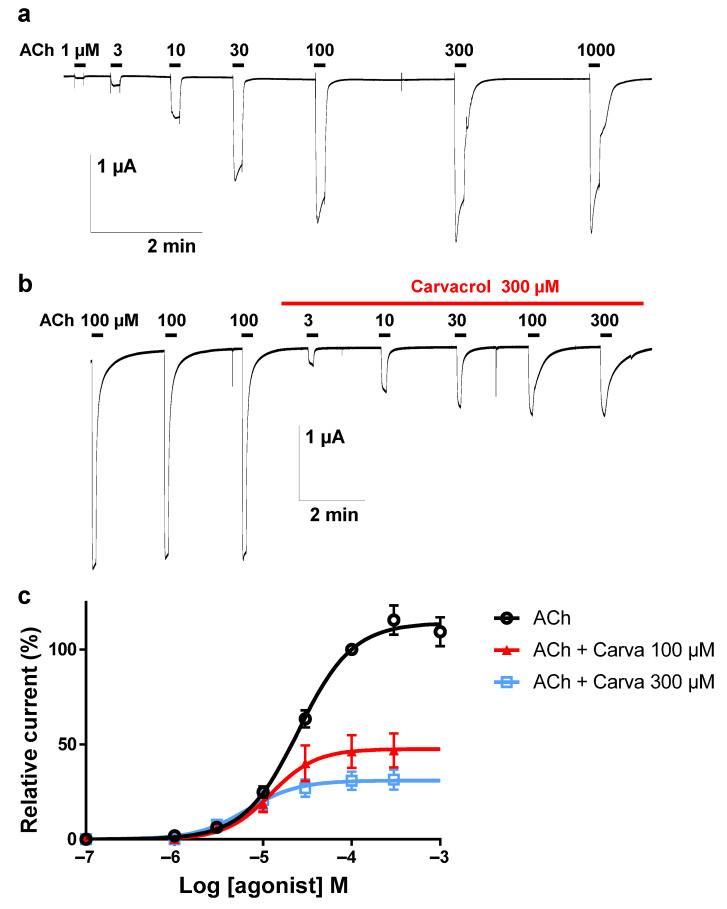
Concentration–response relationships of ACh on the *Parascaris* sp. ACR-26/27 M-AChR expressed in *Xenopus laevis* oocytes in absence of carvacrol (**a**) or in presence of carvacrol (**b**). Representative current traces for single oocytes. The concentrations of ACh and carvacrol (µM) are indicated above each trace. Bars indicate drug applications: ACh was applied for 10 s. (**c**) Concentration–response curves. All responses are normalized to 100 µM ACh. Results are shown as the mean ± SE (*n* = 5–6).

It was recently described that the co-expression of the *Parascaris* sp. ACR-26 and ACR-27 subunits in *Xenopus laevis* oocytes resulted in a functional morantel-sensitive AChR (M-AChR) [29]. The expression of the *Parascaris* 26/27 M-AChR resulted in robust currents in the µA range when challenged with 100 µM acetylcholine (Figure 3a). The ACh EC_50_ value of 25.0 µM (log EC_50_ = 1.398 ± 0.022, *n* = 6) was estimated from the concentration–response curve with current amplitudes normalized to the maximal response to 100 µM (Figure 3c). When carvacrol was perfused in the recording chamber, we observed no agonist action on the M-AChR (Figure 3b). Strikingly, the continued perfusion of 100 and 300 µM carvacrol during the ACh concentration–response relationships significantly decreased the ACh EC_50_ values to 12.2 (log EC_50_ = 1.085 ± 0.064, *n* = 5) and 6.6 µM (log EC_50_ = 0.817 ± 0.060, *n* = 6), respectively (*p* < 0.0001). The Hill coefficients were determined and remained stable in the presence of either 100 (1.7 ± 0.4) or 300 µM carvacrol (1.5 ± 0.3), compared to the absence of carvacrol (1.4 ± 0.1). In the same experiment, we observed that the perfusion of carvacrol significantly reduced the efficacy of ACh activation (I_max_) of this receptor (*p* < 0.0001) (Figure 3b).

**Figure 4:**
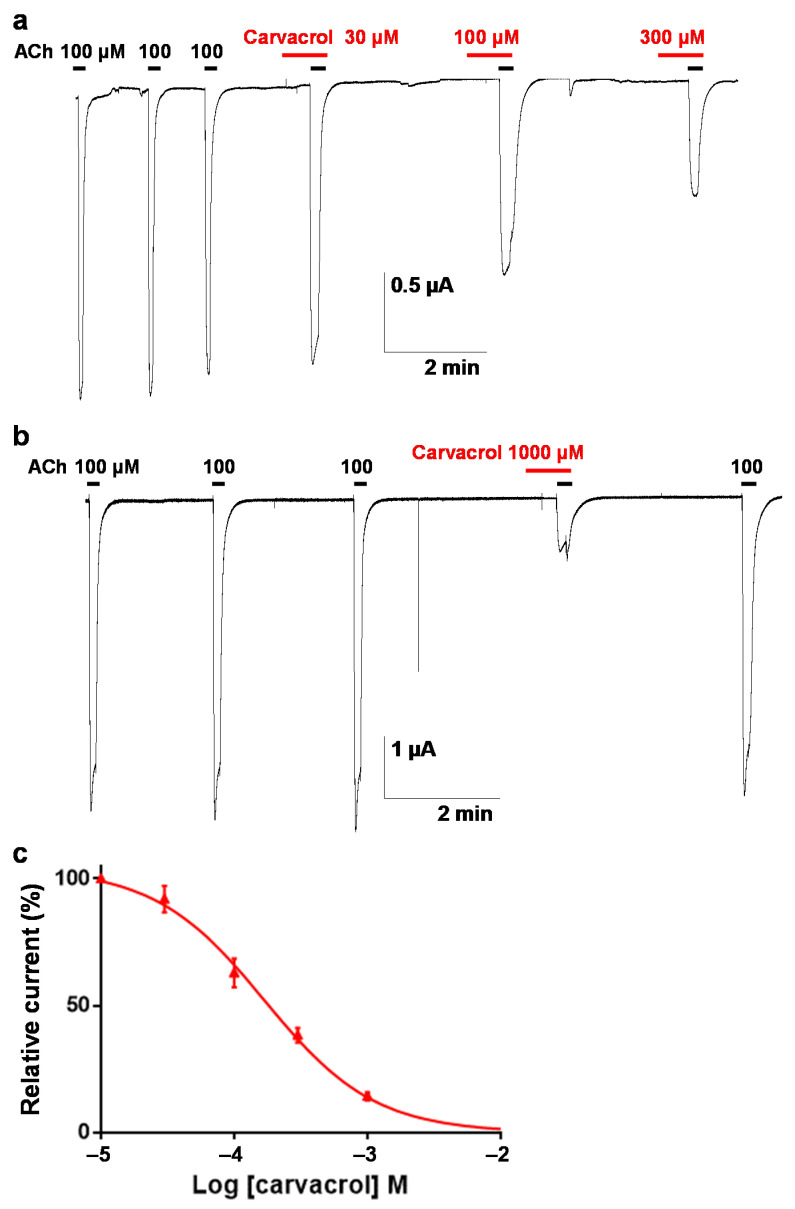
Concentration–inhibition relationship of carvacrol on the *Parascaris* sp. ACR-26/27 M-AChR expressed in *Xenopus* oocytes. Representative current traces for single oocytes challenged with acetylcholine (ACh) in the presence of increasing concentration of carvacrol from 30 to 300 (**a**) and 1000 µM (**b**). The concentrations of ACh and carvacrol (µM) are indicated above each trace. ACh was applied for 10 s (black bars), and carvacrol was applied for 20 s (red bars). (**c**) Concentration–inhibition response curve of carvacrol. All responses are normalized to 100 µM ACh. Results are shown as the mean ± SE (*n* = 7).

To characterize this effect, the carvacrol antagonist concentration–response relationship was obtained by perfusing oocytes with increasing concentrations of carvacrol for 10 s prior to the co-application with 100 µM ACh (Figure 4a,b). Hence, increasing concentrations of carvacrol (10 µM to 1 mM) resulted in a dose-dependent reduction of the maximal ACh-elicited current amplitude. The IC_50_ value of carvacrol for the *Parascaris* M-AChR was 169.3 ± 1.0 µM (*n* = 7) (Figure 4c). Thus, carvacrol slightly increased the ACh affinity for the *Parascaris* M-AChR while acting as a non-competitive antagonist.

### 2.4. Effect of Carvacrol on Parascaris sp. and Ascaris suum Nicotine-Sensitive AChRs Expressed in Xenopus oocytes

**Figure 5:**
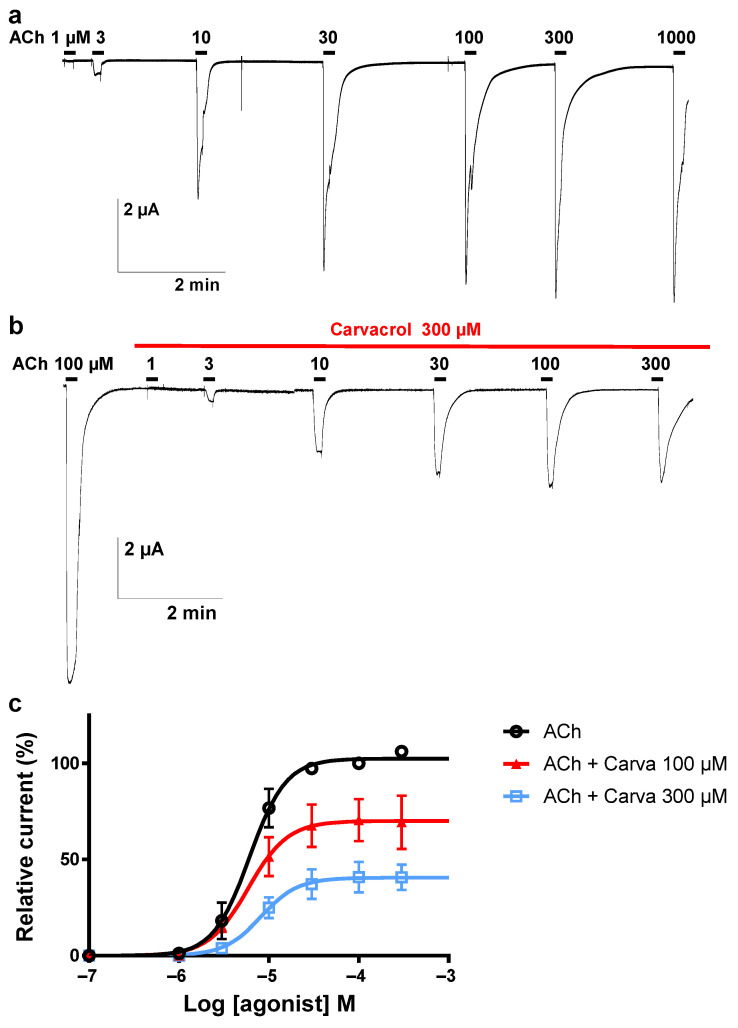
Carvacrol effect on the ACh concentration–response relationships for the *Parascaris* sp. ACR-16 N-AChR expressed in *Xenopus* oocytes in absence of carvacrol (**a**) or in presence of carvacrol (**b**). Representative current traces for single oocytes. The concentrations of ACh and carvacrol (µM) are indicated above each trace. Bars indicate drug applications: ACh was applied for 10 s, and carvacrol was applied for 11 min (red bar). (**c**) Concentration–response curves. All responses are normalized to 100 µM ACh. Results are shown as the mean ± SE (*n* = 6–10).

It was previously reported that the ACR-16 AChR subunit from *Parascaris* sp. and from the closely related species *A. suum* were able to form homomeric functional N-AChRs when expressed in *Xenopus* oocytes [35,36]. Recently, carvacrol proved to be a non-competitive inhibitor of the *A. suum* N-AChR [27]. In order to investigate the mode of action of carvacrol in *Parascaris* sp., we applied carvacrol on oocytes expressing the *Parascaris* sp. N-AChR (Figure 5b). Perfusion of 100 µM acetylcholine elicited large currents with maximum amplitude in the µA range (Figure 5a), and the ACh concentration–response curve was characterized by an EC_50_ of 6.5 µM (log EC_50_ = 0.811 ± 0.028, *n* = 6) (Figure 5c). As expected, a high concentration of carvacrol (300 µM) had no agonist effect (Figure 5b). In the presence of 100 and 300 µM of carvacrol, the EC_50_ values of ACh were 5.9 (log EC_50_ = 0.772 ± 0.052, *n* = 6) and 8.2 µM (log EC_50_ = 0.913 ± 0.038, *n* = 10), respectively, and not significantly different from the ACh EC_50_ obtained without carvacrol (Figure 5c). As for the M-AChR, the Hill coefficients were similar with values of 2.0 ± 0.3, 2.1 ± 0.4 and 2.0 ± 0.2 for 100, 300 µM carvacrol and without carvacrol, respectively. However, the ACh maximal response amplitude was significantly reduced by 2- and 3-fold in the presence of 100 and 300 µM of carvacrol (*p* < 0.05), respectively. Thus, ACh had a lower efficacy as an agonist of the *Parascaris* N-AChR in the presence of carvacrol.

**Figure 6:**
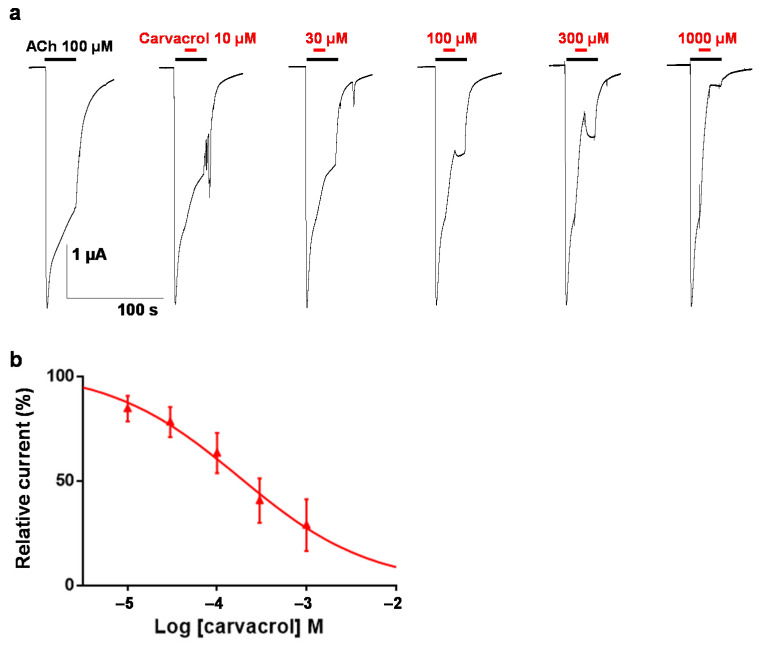
Concentration–inhibition relationship of carvacrol on the *Parascaris* sp. ACR-16 N-AChR expressed in *Xenopus* oocytes. (**a**) Representative current traces for single oocytes challenged with acetylcholine (ACh) in the presence of increasing concentration of carvacrol from 10 to 1000 µM. The concentrations of ACh and carvacrol (µM) are indicated above each trace. ACh was applied for 30 s intervals (black bars), and carvacrol was applied for 10 s (red bars). (**b**) Concentration–inhibition response curve of carvacrol. All responses are normalized to 100 µM ACh. Results are shown as the mean ± SE (*n* = 6).

To characterize this inhibition, carvacrol was perfused during the application of ACh as described elsewhere for different synthetic compounds (Figure 6a) [38]. The carvacrol antagonist concentration–response relationship (10 µM to 1 mM) resulted in a dose-dependent inhibition of the currents with an IC_50_ value of 177.8 ± 1.1 µM (*n* = 6) (Figure 6b). Similarly, we confirmed that 100 µM carvacrol had no impact on the ACh EC_50_ value for the *A. suum* N-AChR (6.0 ± 1.0 (*n* = 6) versus 4.9 ± 1.1 µM (*n* = 11) without carvacrol, *p* > 0.05). In addition, we extended this observation to 300 µM carvacrol (8.9 ± 1.1 µM (*n* = 5)) (Figure S1). As previously described [27], 100 µM carvacrol led to a significant decrease in the ACh maximum response (73.6 ± 1.7%, *p* < 0.05, *n* = 6). Increasing the carvacrol concentration to 300 µM drastically reduced the effect of ACh (19.6 ± 1.5%, *p* < 0.05, *n* = 5). In addition, we determined a carvacrol antagonist dose–response relationship for the *A. suum* N-AChR and obtained an IC_50_ value of 36.4 ± 1.3 µM (*n* = 6) (Figure S2). Altogether, these results indicate that carvacrol acted as a non-competitive antagonist on *Parascaris* sp. and *A. suum* N-AChRs.

## 3. Discussion

There has been limited published data reporting the contraction force transduction in adult parasite worms. In the present study, we carried out for the first time an investigation of *Parascaris* sp. worm pharmacology using contraction assays performed on nerve–muscle preparations. The contractions are not different from the contractions that were obtained in nerve–muscle preparation prepared from *A. suum*, except that the maximal effect is somewhat lower. Indeed, the EC_50_ of ACh from 6.08 µM is similar to the values ranging from 8.87 to 10.88 µM observed in *A. suum* innervated muscle strips [26,37]. These first results indicate that the body muscle flap preparation is a tractable approach to study the pharmacology of the *Parascaris* sp. neuromuscular system. In addition to *A. suum* [39], the measurements of force transduction were described for the sheep barber pole worm *Haemonchus contortus* [40] and the canine hookworm *Ancylostoma caninum*, [41]. Interestingly, these studies provided a better understanding of the diversity of body wall muscle nAChR subtypes that are preferentially activated or antagonized by different cholinergic anthelmintics [42]. In this context, it would be reasonable to expect that the muscle isometric contraction approach could be further adapted for pharmacological investigations in other nematode parasite species of interest such as the ascarids *Taxocara canis*, *Ascaridia galli* and *Anisakis simplex*. Furthermore, when anthelmintic-resistant parasites were available, the comparison of muscle contraction assays with drug-susceptible nematode parasites revealed new insights into the mechanisms underpinning resistance to anthelmintics [40,41]. As little is known on the effect of cholinergic anthelmintics on *Parascaris* sp. Muscles, the muscle contraction approach will be useful for to assess the nAChRs present in *Parascaris* sp. and the changes that could be associated with resistance. In *C. elegans*, *A. suum* and the pig nodule worm *Oesophagostomum dentatum*, single channel recordings revealed at least three main nAChR subtypes characterized by their conductance [43,44,45]. Likewise, single channel experiments in somatic muscle cells of *Parascaris* sp. could be helpful to investigate the muscle nAChR subtypes targeted by anthelmintics and carvacrol in vivo.

Given the limited number of anthelmintic drugs available for the control of *Parascaris* sp. infestations (benzimidazole, pyrantel, ivermectin and moxidectin) and the growing issue of anthelmintic resistance worldwide, there is an urgent need to develop new alternative control strategies [7,10]. Hence, increasing attention is given to the nematocidal potential of plant-based natural products [46], including essential oils, which could replace or potentiate the effects of classical anthelmintic drugs [12]. The advantage of this approach is the possibility of continuous application of functional feeds, thus preventing reinfection after deworming, which does not provide long-term protection against infection. Among the active ingredients from essential oils, carvacrol was shown to be active against animal parasitic nematodes, plant parasitic nematodes and the free-living nematode *C. elegans* [18,22,46]. Here, we took advantage of the adapted neuromuscular contraction approach to assess the effect of carvacrol in *Parascaris* sp. We found that carvacrol completely abolished the contractions induced by ACh, and this effect remained even after removal of carvacrol from the experimental bath. Based on this result, we hypothesized that carvacrol may interact directly with nAChRs. We would like to comment on the fact that after incubation of the neuromuscular flaps with 300 µM of carvacrol, it was not possible to obtain contractions again. In our previous studies on *A. suum*, the effect was reversible, and contractions almost reached the control value after washing. Given the results obtained after receptor expression on oocytes, we hypothesize that the reason for this nature of carvacrol action is the anatomical and morphological specificity of *Parascaris* sp. that we observed. Namely, we assume there may be a kind of cumulative effect of carvacrol and the impossibility of its removal by washing. The body wall of *Parascaris* sp. is 2–3 mm thicker than in *A. suum*, due to the three-layer collagen sheath that holds the carvacrol and makes it impossible to wash. This assumption should certainly be examined in future research.

Our electrophysiological investigations demonstrated the non-competitive inhibition of carvacrol on both the nicotine-sensitive ACR-16 and the morantel-sensitive ACR-26/27 AChRs from *Parascaris* sp. expressed in *Xenopus* oocytes. In addition, this effect was further confirmed for the ACR-16 N-AChR from *A. suum*, which is closely phylogenetically related to *Parascaris* sp. This not the first time that carvacrol has been assayed on *A. suum* nAChRs. It was previously observed that carvacrol produced significant inhibition of *A. suum* muscle contractions induced by ACh, inhibited depolarizations caused by acetylcholine and reduced membrane conduction of muscle cells [26]. Unlike menthol, carvacrol has further been reported to produce non-competitive inhibition on the *A. suum* ACR-16 N-AChR [27]. More recent contraction experiments revealed the antagonistic interaction of carvacrol with anthelmintic drugs at different muscle nicotinic receptors in vivo [28]. Interestingly, the full inhibition of the ACh contractile effect with 300 µM of carvacrol was markedly different from the effect in *A. suum*, which did not exceed 49% [26,28]. This result suggests that *Parascaris* worms may be more sensitive to carvacrol than Ascaris worms. On the other hand, our data are consistent with the results for *A. suum* ACR-16 N-AChRs, in which carvacrol acted as a non-competitive antagonist [27]. In addition, we further confirmed this effect on *Parascaris* sp. ACR-16 N-AChRs and extended to the ACR-26/27 M-AChRs. However, according to our concentration–inhibition data, carvacrol showed approximately 5-fold higher affinity for the *A. suum* N-AChR over the *Parascaris* sp. N -AChR. Therefore, it is not possible to rule out that additional mechanisms may be involved in the activity of carvacrol in *Parascaris* sp.

Noticeably, carvacrol and cinnamaldehyde showed a better potency in multi-drug resistant *H. contortus* egg hatch assay when combined together, and this result highlights the anthelmintic value of bioactive compounds from plant sources [20]. However, the literature is scarce on the clinical efficacy of herbal essential oils either alone or in combination with synthetic drugs in vivo, whereas numerous studies have shown interesting effects in vitro. Some recent investigations on plant product combination with anthelmintic drugs have reported potentially interesting synergistic effects against gastrointestinal parasites [47,48,49]. The potential of carvacrol and essential oils either alone or in association with anthelmintic drugs in treating *Parascaris* sp. infections in equids remains to be evaluated.

## 4. Materials and Methods

### 4.1. Parascaris sp. Muscle Flap Contraction

For the contraction assay, adult female *Parascaris* sp. worms were collected weekly from the slaughterhouse at Vrčin, Belgrade, Serbia. Worms were maintained in Locke’s solution, composition (mM): NaCl 155, KCl 5, CaCl_2_ 2, NaHCO_3_ 1.5 and glucose 5, at a temperature of 32 °C. The Locke’s solution was changed twice daily, and each batch of worms was used within 2 days of collection. *Parascaris* muscle flaps for the contractions were prepared by dissecting the anterior part of the worm, 3–4 cm caudal to the head (Figure 1b). Each flap (always the same length of 1 cm) was monitored isometrically by attaching a force transducer in an experimental bath maintained at 37 °C, containing 20 mL Ascaris Perienteric Fluid Ringer/APF Ringer (mM: NaCl, 23; Na-acetate, 110; KCl, 24; CaCl_2_, 6; MgCl_2_, 5; glucose, 11; HEPES, 5; pH 7.6) and bubbled with room air. After dissection, the preparations were allowed to equilibrate for 15 min under an initial tension of 1.5 g. Different concentrations of ACh were then added to the preparation (1, 3, 10, 30 and 100 μM), and the maximum contraction was observed before washing and subsequent application of the next concentration of acetylcholine. Responses for each concentration were expressed in grams of tension (g), produced by each individual flap preparation. The effect of carvacrol (300 μM) on the acetylcholine dose–response plots was determined. Contractions were monitored on a PC using a BioSmart interface and eLAB software (ElUnit, Belgrade, Serbia). The system allows real-time recording, display and analysis of experimental data. Sigmoid dose–response curves for each individual flap preparation at each concentration of the antagonist were described by the Hill equation.

### 4.2. Two-Electrode Voltage-Clamp Electrophysiology in Xenopus laevis oocytes

*Parascaris* sp. ACR-26/27 M-AChR as well as *Parascaris* sp. and *A. suum* ACR-16 N-AChRs were expressed in *Xenopus laevis* oocytes as previously described [29,35,36]. Briefly, *Xenopus laevis* defolliculated oocytes were obtained from Ecocyte Bioscience (Germany). Oocytes were micro-injected with 36 nL of cRNA mixes containing 50 ng/µL of each cRNA encoding subunits of interest and three *H. contortus* ancillary factors (Hco-RIC-3.1, Hco-UNC-50 and Hco-UNC-74). After 3–4 days of incubation, the oocytes were assayed under voltage clamp at −60 mV, and electrophysiological recordings were performed as described previously. The carvacrol concentration-dependent inhibition of acetylcholine current response was assessed for *Parascaris*- and Asu-ACR-16 channels with the protocol described by Zheng et al. [38].

### 4.3. Drugs

Acetylcholine chloride (ACh) and carvacrol were purchased from Sigma-Aldrich.

### 4.4. Statistical Analyses

The results of the contraction assay are expressed as means ± S.E. in grams (g) of tension. Sigmoid concentration dose–response is described by the equation as follows: % response = 1/1 + [EC50/Xa] nH, where the median effective concentration (EC_50_) is the concentration of the agonist (Xa) producing 50% of the maximum response, and nH is the Hill coefficient (slope). GraphPad Prism^®^ Software (San Diego, CA, USA) was used to estimate the constants EC_50_ and nH by non-linear regression for each preparation. We determined the mean contraction responses to each concentration of acetylcholine. Whole cell current electrophysiology responses were analyzed using the pCLAMP 10.4 package (Molecular Devices). EC_50_ and IC_50_ values were determined using non-linear regression on normalized data (100 µM ACh as maximal response) using GraphPad Prism^®^ software. One-way analysis of variance (ANOVA) was applied for the comparison of the differences between the EC_50_ value and the maximal effect (R_max_). Differences were considered significant when the *p* value was < 0.05. The statistical analysis was conducted using GraphPad Prism^®^ software (San Diego, CA, USA), while all values are expressed as mean ± standard error (S.E.).

## 5. Conclusions

In summary, we report for the first time in vivo contraction assays from *Parascaris* sp. neuromuscular preparation. Our findings indicate that the antimicrobial agent carvacrol inhibited nAChR function in vivo on *Parascaris* sp. muscle contractions and in vitro on both morantel- and nicotine-sensitive nAChRs. The present study improves the understanding of the anthelmintic mode of action of plant essential oil ingredients and opens the way for new therapeutic prospects in equine medicine.

## Data Availability

Data sharing not applicable.

## Supplementary Materials

The following are available online at https://www.mdpi.com/article/10.3390/ph14060505/s1, Figure S1: Carvacrol effect on the acetylcholine concentration–response relationships for the *Ascaris suum* ACR-16 N-AChR expressed in *Xenopus* oocytes, Figure S2: Concentration–inhibition relationship of carvacrol on the *A. suum* ACR-16 N-AChR expressed in *Xenopus* oocytes.
