# Supplementary material for: Action of Carvacrol on Parascaris sp. and Antagonistic Effect on Nicotinic Acetylcholine Receptors
Source: Pharmaceuticals (Basel). 2021 May 26;14(6):505. doi: 10.3390/ph14060505 (PMC8226574; doi:10.3390/ph14060505)
Supplement: Supplementary file 1 [file pharmaceuticals-14-00505-s001.zip › pharmaceuticals-1215551-supplementary.pdf]

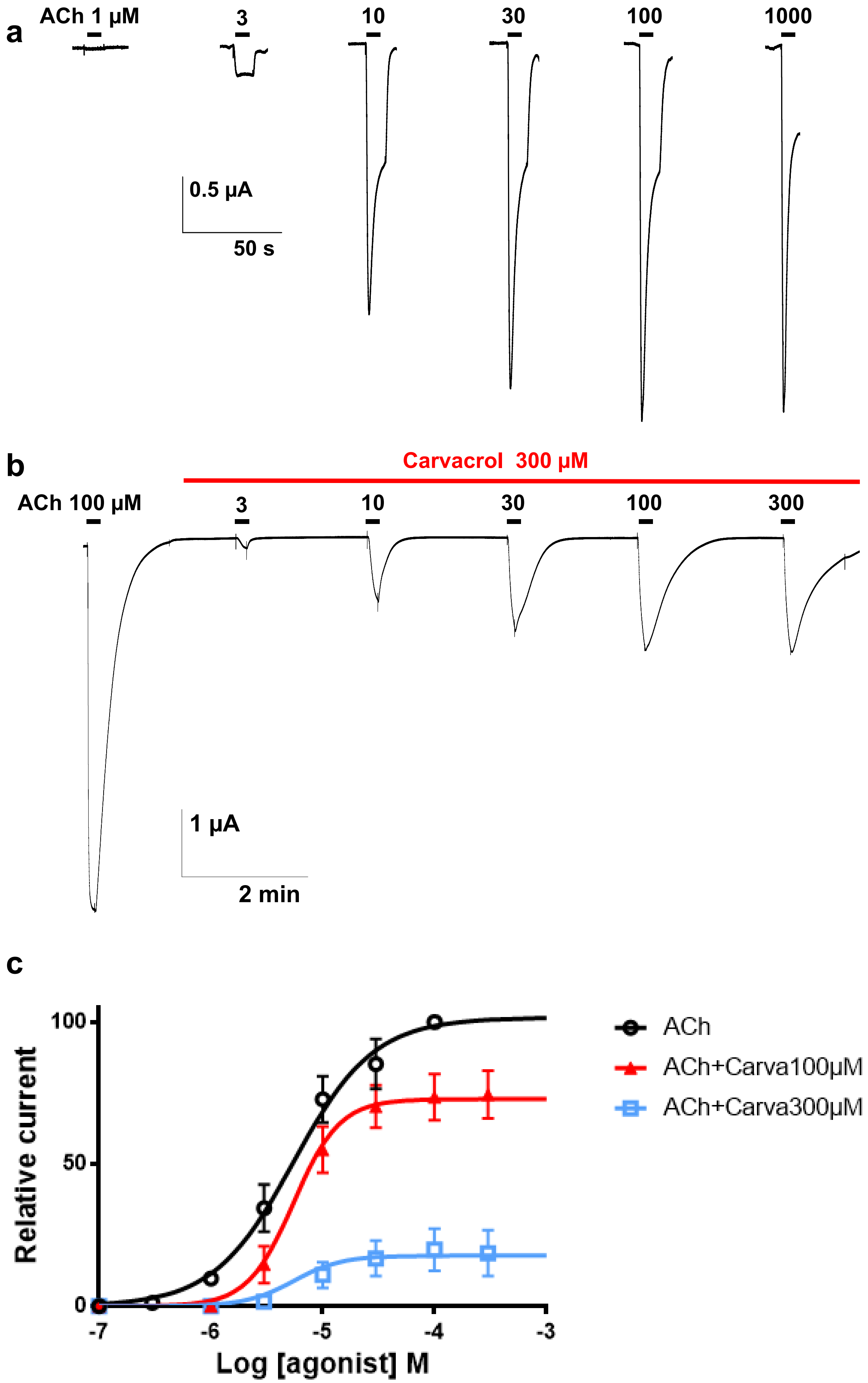

Figure S1 Carvacrol effect on the acetylcholine concentration-response relationships for the *Ascaris suum* ACR-16 N-AChR expressed in *Xenopus* oocytes. (**a**) Recordings of ACh-evoked currents in absence of carvacrol; (**b**) Recordings of ACh-evoked currents in presence of 300 µM carvacrol; (**c**) Concentration-response curves. All responses are normalized to 100 µM ACh. Results are shown as the mean ± se.

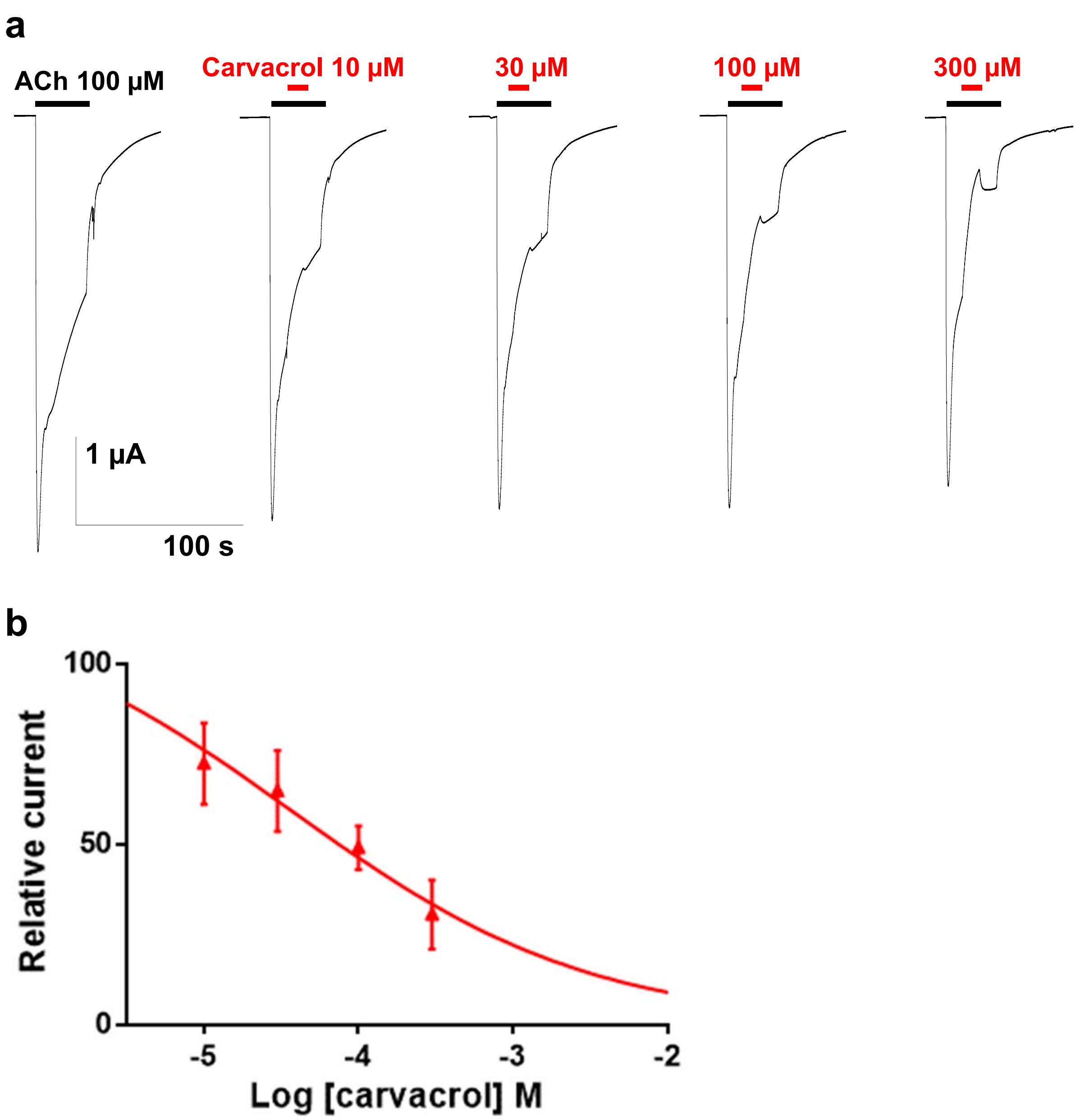

Fig. S2 Concentration-inhibition relationship of carvacrol on the *A. suum* ACR-16 N-AChR expressed in *Xenopus* oocytes. (**a**) Representative current traces for single oocytes challenged with acetylcholine (ACh) in the presence of increasing concentrations of carvacrol. The concentrations of ACh and carvacrol (μM) are indicated above each trace; (**b**) Concentration-inhibition response curves of carvacrol. All responses are normalized to 100 μM ACh. Results are shown as the mean ± se.
